# Soil bacterial communities and their associated functions for forest restoration on a limestone mine in northern Thailand

**DOI:** 10.1371/journal.pone.0248806

**Published:** 2021-04-08

**Authors:** Chakriya Sansupa, Witoon Purahong, Tesfaye Wubet, Pimonrat Tiansawat, Wasu Pathom-Aree, Neung Teaumroong, Panuwan Chantawannakul, François Buscot, Stephen Elliott, Terd Disayathanoowat

**Affiliations:** 1 Department of Biology, Faculty of Science, Chiang Mai University, Chiang Mai, Thailand; 2 Graduate School, Chiang Mai University, Chiang Mai, Thailand; 3 Department of Soil Ecology, UFZ-Helmholtz Centre for Environmental Research, Halle (Saale), Germany; 4 Department of Community Ecology, UFZ-Helmholtz Centre for Environmental Research, Halle (Saale), Germany; 5 German Centre for Integrative Biodiversity Research (iDiv), Halle-Jena-Leipzig, Leipzig, Germany; 6 Environmental Science Research Centre and Forest Restoration Research Unit, Biology Department, Science Faculty, Chiang Mai University, Chiang Mai, Thailand; 7 School of Biotechnology, Institute of Agricultural Technology, Suranaree University of Technology, Nakhon Ratchasima, Thailand; 8 Research Center in Bioresources for Agriculture, Industry and Medicine, Chiang Mai University, Chiang Mai, Thailand; 9 Research Center of Microbial Diversity and Sustainable Utilization, Chiang Mai University, Chiang Mai, Thailand; Government College University Faisalabad, PAKISTAN

## Abstract

Opencast mining removes topsoil and associated bacterial communities that play crucial roles in soil ecosystem functioning. Understanding the community composition and functioning of these organisms may lead to improve mine-rehabilitation practices. We used a culture-dependent method, combined with Illumina sequencing, to compare the taxonomic richness and composition of living bacterial communities in opencast mine substrates and young mine-rehabilitation plots, with those of soil in adjacent remnant forest at a limestone mine in northern Thailand. We further investigated the effects of soil physico-chemical factors and ground-flora cover on the same. Although, loosened subsoil, brought in to initiate rehabilitation, improved water retention and facilitated plant re-establishment, it did not increase the population density of living microbes substantially within 9 months. Planted trees and sparse ground flora in young rehabilitation plots had not ameliorated the micro-habitat enough to change the taxonomic composition of the soil bacteria compared with non-rehabilitated mine sites. Viable microbes were significantly more abundant in forest soil than in mine substrates. The living bacterial community composition differed significantly, between the forest plots and both the mine and rehabilitation plots. Proteobacteria dominated in forest soil, whereas Firmicutes dominated in samples from both mine and rehabilitation plots. Although, several bacterial taxa could survive in the mine substrate, soil ecosystem functions were greatly reduced. Bacteria, capable of chitinolysis, aromatic compound degradation, ammonification and nitrate reduction were all absent or rare in the mine substrate. Functional redundancy of the bacterial communities in both mine substrate and young mine-rehabilitation soil was substantially reduced, compared with that of forest soil. Promoting the recovery of microbial biomass and functional diversity, early during mine rehabilitation, is recommended, to accelerate soil ecosystem restoration and support vegetation recovery. Moreover, if inoculation is included in mine rehabilitation programs, the genera: *Bacillus*, *Streptomyces* and *Arthrobacter* are likely to be of particular interest, since these genera can be cultivated easily and this study showed that they can survive under the extreme conditions that prevail on opencast mines.

## Introduction

Opencast mining is one of the most common methods of mineral extraction [1]. Although, the method is important for social and economic development, it has severe negative effects on the environment [2–4], particularly removal of topsoil and vegetation. Consequently, mined sites are classified as the severest form of land degradation [5], requiring an intensive array of rehabilitation procedures to recover biomass, ecosystem structure, biodiversity, ecological functioning and environmental services after mine-closure [6]. Thus far, mine rehabilitation has mostly focused on the recovery of plant and animal communities above-ground [7–10]. The soil ecosystem and its microbial inhabitants have received less attention, even though they play important roles in ecosystem functions and services [11].

Soil bacteria drive numerous biochemical cycles [12], improve soil structure, and increase nutrient availability [11]. They also enhance plant nutrient uptake, control pathogens and increase stress resilience [13]. Such functions can affect rehabilitation outcome. Several studies show that microbial community composition can be used as an indicator of rehabilitation progress [14–16]. However, information about the composition of microbial communities that persist in mine substrates and how they might both influence and respond to mine rehabilitation, in its early stages, is still scarce. Inoculation of mine substrates with bacteria, to enhance rehabilitation, is being actively investigated, to improve plant productivity [17–19] post mine closure. However, few bacteria inocula can survive in mine substrates [18]. The taxonomic and functional composition of soil bacterial communities change along a rehabilitation chronosequence [15, 20, 21]. The fact that bacterial taxa that are dominant in mined and early rehabilitation substrates are rare in late-successional forest, suggests that only a specific group can perform well in the immediate post-mining environment [15, 22]. Consequently, information about which taxa can persist on mines is crucial, to support mine restoration initiatives.

In this study, we investigated the taxonomic and functional diversity of living soil bacteria communities at an opencast limestone mine in northern Thailand. Specifically, we isolated bacteria from mine substrates (both mined and rehabilitation sites) and from adjacent forest soil, using a conventional culturing method and subsequently applied next-generation sequencing (NGS) to determine taxonomic diversity. Moreover, we determined responses of the soil bacteria community to soil physico-chemical properties and ground-flora cover. We tested the hypotheses that i) some bacteria taxa can persist in mine substrates, despite severe degradation [23], ii) living bacterial communities and their functional composition differ significantly among three soil/substrates (forest soil, post-mining substrate and loosened subsoil used to initiate rehabilitation) and iii) soil physico-chemical variables and vegetation cover correlate with the taxonomic and functional composition of the soil bacterial community [15, 24].

## Materials and methods

### Study site, experimental design and sample processing

The study site was the Siam Cement Group’s semi-opencast limestone mine in Lampang Province, northern Thailand (18°32´23´´ N, 99°34´47´´ E), surrounded by natural bamboo-deciduous forest [25]. The mine floor was hard-packed, exposed, rock and gravel. In small plots around the main pit perimeter, rehabilitation procedures had been implemented for 9 months. These involved covering the mine substrate with sub-soil (brought in from a stockpile within the mine concession) and planting saplings of tree species that are characteristic of the surrounding forest, selected for their ability to enhance forest regeneration (i.e. the framework tree species) [9].

**Fig 1:**
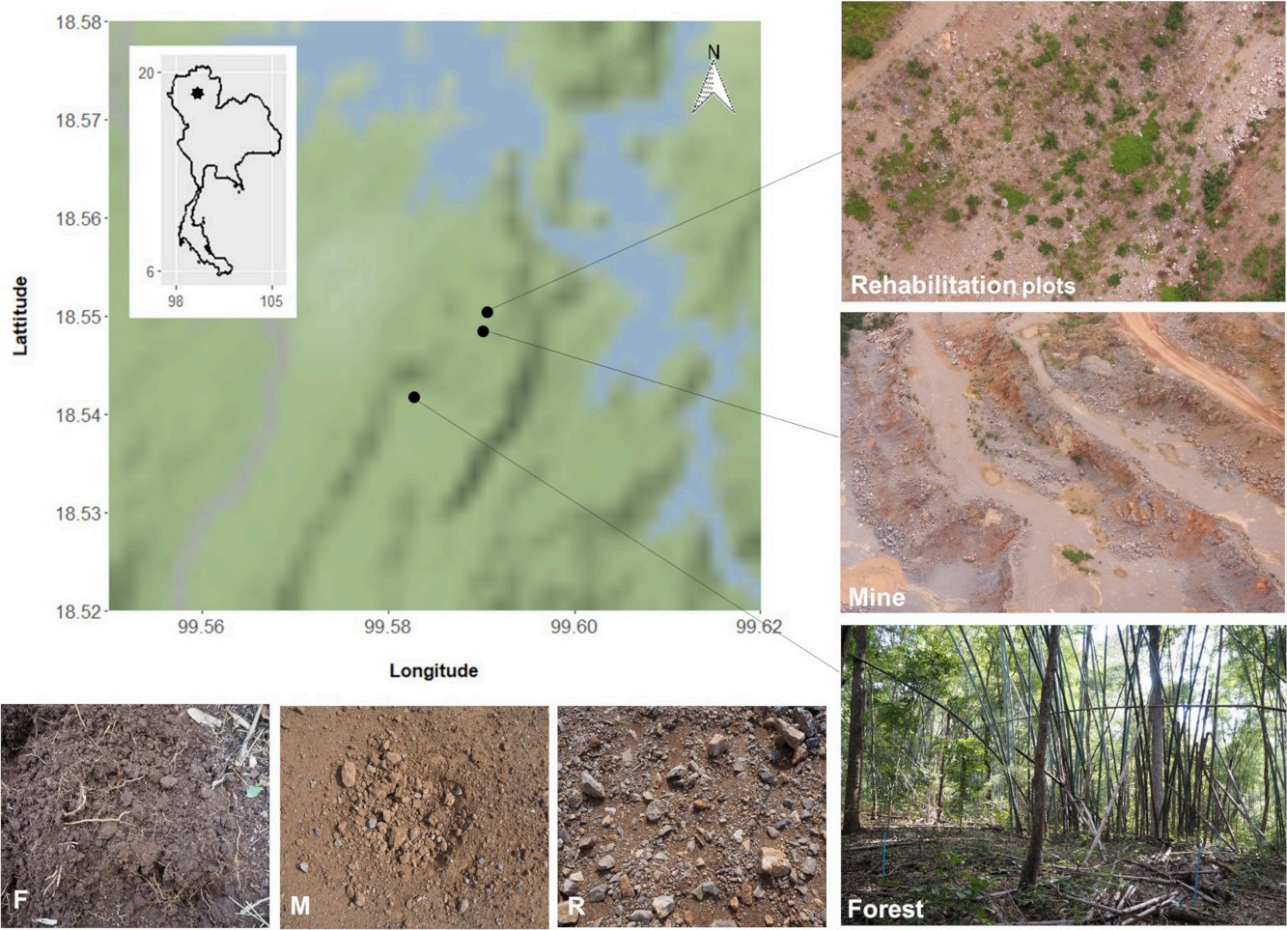
Study sites. Bottom left showing three contrasting soil/substrates from forest (F), mine (M) and young mine-rehabilitation plots (R). Map tiles by Stamen Design, under CC BY 3.0. Data by OpenStreetMap, under ODbL.

Three substrates were sampled: i) soil from bamboo-deciduous forest adjacent to the mine (F), ii) substrate from the mine floor (M) and iii) sub-soil that had been layered on top of the mine substrate during rehabilitation procedure (R)–finer and looser than the mine-floor substrate, but retaining small rocks and without the organic matter that is characteristic of topsoil. The latter had been planted with saplings of framework tree species (30–50 cm tall) 9 months before sample collection (Fig 1).

Substrate/soil samples were collected in June 2018. Five replicated plots, (5x5 m) >20 m apart, were selected on each of the 3 sites, in and around the mine. Five samples were collected to 10-cm depth in each plot, using an auger 10 cm in diameter. The samples were bulked into one composite sample and passed through a 2-mm sieve to remove stones, roots and organic litter [26]. The samples were kept in an icebox during transportation.

### Measuring soil physico-chemical properties and vegetation cover

Soil pH (H_2_O and 0.01 M CaCl_2_) were measured, using well-established standard procedures [27]. Soil moisture, organic matter (SOM), texture (sand, slit, clay), inorganic nutrients (total nitrogen (N), phosphorous (P), potassium (K), calcium (Ca), magnesium (Mg), iron (Fe), manganese (Mn), sulfur (S) and boron (B)) and cation exchange capacity (CEC), were measured, using the protocols in S1 Table. All physico-chemical analyses were conducted in triplicate for each composite sample. Six plant variables, including: percent cover of grasses, herbs and shrubs, total ground-flora cover, tree crown cover and plant species richness, were determined over each of the replicate plots.

### Direct extraction of soil DNA

DNA was first extracted from 0.25g of soil using a NucleoSpin® Soil kit, according to the manufacturer’s protocols. DNA concentration and purity were measured using a NanoDrop spectrophotometer. Subsequently, the DNA was amplified by polymerase chain reaction (PCR) targeted at the 16S rRNA gene. Two pair of universal primers, including Bact341F (5’-CCTACGGGNGGCWGCAG-3’)—Bact785R (5’-GACTACHVGGGTATCTAATCC-3’) [28] and 27F (5´-AGAGTTTGATCMTGGCTCAG-3´) - 1492R (5´-TACGGYTACCTTGTTACGACT-3´) [29], were used to confirm the presence of bacteria. Amplifications of the former (Bact341F-Bact785R) was performed in 25 μL reactions with Qiagen HotStar Taq master mix (Qiagen Inc, Valencia, California), 1 μL of each 5 μM primer, and 1 μL of template. PCR reaction was performed on ABI Veriti thermocyclers (Applied Biosytems, Carlsbad, CA, USA) under the following thermal profiles: 95^○^C for 15 min, then 35 cycles of 94^○^C for 30 sec, 55^○^C for 30 sec, 72^○^C for 1 min, followed by one cycle of 72^○^C for 10 min. On the other hand, amplification of the latter (27F-1492R) was performed in the following PCR mixture (25 μL total volume): 1X PCR buffer (10 mM Tris-HCl, pH 8.3 at 25°C, 50 mM KCl), 0.2 mM dNTP Mix (a mixture of four nucleotides: dATP, dTTP, dCTP, dGTP), 1.5 mM MgCl_2_, 0.8 μM each of the primers, 1 unit of Taq DNA polymerase (5 U/μL), 1 μL of DNA template and sterile deionized water, adjusting the final volume to 25 μL. The following PCR cycle was used: an initial denaturation of 94°C for 3 min, followed by 30 cycles of 94°C for 45 secs, 55°C for 30 secs and 72°C for 90 secs, and a final extension of 72°C for 10 min. The PCR product was visualized on 1.5% (w/v) agarose gels. Using these procedures, we did not get high quality DNA from the substrates of mine and rehabilitation plot. Therefore, a OneStep PCR Inhibitor Removal Kit (Zymo Research) was used to remove the PCR inhibitor. Furthermore, two other DNA extraction kits (Quick-DNA Fecal/Soil Microbe Miniprep Kit—Zymo Research and PowerSoil® DNA Isolation Kit—Qiagen) and DNA extraction protocol of Direito et al. [30] and Aoshima et al. [31] were applied to extract DNA from the samples. Subsequently, the DNA from these additional extraction protocols was amplified and visualized, as described above. Finally, the DNA samples from forest soil were sent to Macrogen, South Korea to perform Illumina MiSeq sequencing. Sequencing protocol and bioinformatics analysis of the samples were explained in S1 Text.

### Investigating bacterial diversity, community composition and ecological functioning

#### Culturing soil bacteria

Soil bacteria were cultivated within 24 h after sampling. One gram of soil was added to 9 ml of 0.85% NaCl and mixed thoroughly to homogenize the soil suspension. Subsequently, 100 μl of the soil suspension was cultured on 3 different culture media: i) plate count agar (PCA), ii) nutrient agar (NA) and iii) tryptone soy agar (TSA), using 2 methods: i) the pour-plate technique whereby suspensions were introduced onto empty plates, before adding melted agar and gently swirling the plates to mix the samples with the agar and ii) the spread-plate technique whereby soil suspensions were added to a solidified medium and spread over the plates. The latter were incubated under both aerobic and anaerobic conditions. The use of various techniques and conditions allowed us to detect the most possible living bacteria in the samples. Subsequently, all plates were incubated at 25°C for 3 days, after which the number of colonies on each plate was counted and the number of colony-forming units per gram of dry soil (CFU/gdw) was calculated. Finally, all colonies, derived from each sample type, were collected and mixed in collection tubes. These mixed colony suspensions were stored at -20°C for subsequent DNA analyses.

#### Identifying soil bacteria taxa and deriving their functions by DNA extraction, sequencing and bioinformatics

We used the mixed colony suspensions for analysis of bacterial genomic DNA, instead of directly extracted soil DNA (eDNA). This method is appropriate for fast screening of living bacteria and their associated function. Bacterial genomic DNA was extracted from 300 μL of each colony suspension, using a NucleoSpin® Soil DNA extraction kit, following the manufacturer’s instructions. Genomic DNA samples were then kept at -20°C, until further analyses. To determine bacterial sequences, all genomic DNA was analyzed, using paired-end Illumina Miseq, following the manufacturer’s instructions. Briefly, the sequencing targets 16S ribosomal RNA V3—V4 region was performed, using the forward primer: Bact341F (5’-CCTACGGGNGGCWGCAG-3’) and the reverse primer: Bact785R (5’-GACTACHVGGGTATCTAATCC-3’) [28]. For library preparation, Illumina-adapter-overhang nucleotide sequences were added to the primers. Amplification was performed in the following PCR mixture (total volume 25 μL): 12.5 μL of 2X KAPA HiFi HotStart ReadyMix, 2.5 μL of each 1 μM primer and 2.5 μL (5 ng/μL) of DNA template. The PCR reaction was performed as follows: denaturing at 95°C for 3 min, followed by 25 cycles of denaturing at 95°C for 30 secs, annealing at 55°C for 30 secs and extension at 72°C for 30 secs, and a final extension at 72°C for 5 min. The PCR amplicons were then cleaned up and prepared for sequencing. The library was sequenced on an Illumina MiSeq platform at Macrogen, South Korea. Eventually, raw sequence/read datasets of each sample (total 14 datasets: F = 5, M = 5, R = 4) were generated and used to identify the bacterial taxa with bioinformatics analysis. The raw sequences for this study can be found at Sequence Read Archive (SRA) database of National Center for Biotechnology Information (NCBI), under BioProject number: PRJNA548272.

For bioinformatics analysis, individual sample raw sequences/reads were analyzed using MOTHUR 1.33.3 [32] and the Standard Operating Procedure (SOP) custom-analysis workflow [33]. Briefly, raw reads, which had overlapping sequences of ≥ 20 base pairs, were first assembled to generate paired-end reads. The paired-ending was followed by quality filtering for high-quality reads (length ≥ 200 base pairs, Phered score ≥ 30, probability of incorrect base called ≤ 0.1%). Chimeric sequences were detected, using the UCHIME algorithm [34], as implemented in MOTHUR, and removed. The cleaned sequences were clustered at 97% sequence identity, into groups called Operational Taxonomic Units (OTU). Representative sequences of each OTU were used to assign taxonomy, using the SILVA 16S rRNA sequence database version 128 [35]. OTUs with 3 reads or lower (singletons, doubletons, and tripletons) were removed to eliminate potential sequencing errors. The remaining sequence dataset was then rarefied to 30,000 reads per sample. Finally, an OTU abundance table was created, containing assigned taxonomic OTUs and the number of sequences per individual OTU in each sample.

Furthermore, all bacterial OTUs from normalized data were used to predict ecological function, using the database: Functional Annotation of Prokaryotic Taxa or FAPROTAX [36], which assigns ecological functions to bacterial taxa based on published accounts of bacterial metabolism. The database covers more than 4,600 taxa and 80 functions. Although FAPROTAX was first created for marine ecosystems, recent publications indicate that it can also be applied to a variety of other ecosystems, including soil ecosystems [37–39].

### Statistical analyses

#### Differences in soil/substrate properties and vegetation among sampling sites

Data from the F, M and R samples were analyzed as follows: 17 variables, related to physico-chemical conditions, and 6 related to vegetation cover were associated with each sample. A one-way analysis of variance (One-way ANOVA) was performed to test for differences in substrate/soil physico-chemical conditions among the three sites for 12 variables that met the requirements of ANOVA (normal distribution). For the other 5 physico-chemical variables, differences among sites were tested by the Kruskal-Wallis test (Table 1). Vegetation cover on the mine was zero. Consequently, vegetation cover comparisons were limited to the F and R sample sites, using t-tests. All tests were performed using PAST software [40].

**Table 1:** Physico-chemical properties of soil/substrate and plant variables.

Variables	F	M	R	Statistical test
**Soil variables**				
pH (H_2_O)	7.19 ± 00.13a	8.82 ± 00.14b	8.55 ± 00.05ab	Kruskal-Wallis
pH (CaCl_2_)	6.59 ± 00.18a	7.89 ± 00.01b	7.73 ± 00.01b	One-way ANOVA
Moisture (%)	23.34 ± 00.43c	2.11 ± 00.37a	4.99 ± 00.82b	One-way ANOVA
SOM (%)	6.65 ± 00.03c	0.42 ± 00.07a	0.94 ± 00.25b	One-way ANOVA
Total N (%)	0.33 ± 00.00b	0.02 ± 00.00a	0.05 ± 00.01a	Kruskal-Wallis
P (mg/kg)	126.01 ± 55.43b	0.49 ± 00.00a	3.44 ± 02.95ab	Kruskal-Wallis
K (mg/kg)	326.66 ± 56.71b	33.20 ± 06.99a	72.57 ± 13.94a	One-way ANOVA
Ca (mg/kg)	5459.38 ± 478.96a	4941.73 ± 59.75a	5964.54 ± 258.72a	One-way ANOVA
Mg (mg/kg)	350.80 ± 19.90b	98.90 ± 08.35a	189.32 ± 43.14a	One-way ANOVA
Fe (mg/kg)	18.94 ± 04.26b	2.97 ± 00.36a	4.81 ± 00.70a	One-way ANOVA
Mn (mg/kg)	155.69 ± 38.75b	9.05 ± 00.84a	24.08 ± 07.13a	One-way ANOVA
S (mg/kg)	12.99 ± 00.53a	10.89 ± 04.84a	2.60 ± 00.71a	Kruskal-Wallis
B (mg/kg)	0.05 ± 00.01b	0.19 ± 00.00a	0.19 ± 00.00a	Kruskal-Wallis
CEC (cmol/kg)	37.88 ± 02.43b	5.07 ± 00.91a	11.14 ± 02.26a	One-way ANOVA
Sand (%)	10.56 ± 01.17a	64.42 ± 01.73c	47.24 ± 03.87b	One-way ANOVA
Slit (%)	12.80 ± 01.74a	17.60 ± 01.33a	18.00 ± 01.63a	One-way ANOVA
Clay (%)	77.44 ± 00.75c	17.98 ± 01.07a	34.76 ± 02.89b	One-way ANOVA
**Plant variables**				
Grasses (%)	8.00 ± 02.00b	0.00 ± 00.00	3.00 ± 02.35a	t-test
Herbs (%)	52.00 ± 08.60b	0.00 ± 00.00	3.25 ± 02.25a	t-test
Shrubs (%)	8.00 ± 01.22b	0.00 ± 00.00	0.25 ± 00.25a	t-test
Total ground-flora cover (%)	60.00 ± 09.62b	0.00 ± 00.00	8.75 ± 03.75a	t-test
Tree crown cover (%)	74.00 ± 14.00b	0.00 ± 00.00	0.00 ± 00.00a	t-test
No. of plant species	21.20 ± 01.16b	0.00 ± 00.00	11.75 ± 01.11a	t-test

Measuring from forest soil (F), mine substrate (M) and young mine-rehabilitation substrate (R).

#### Differences in community taxonomic composition and functional groups abundance among sampling sites

The OTU richness and rarefaction curves of each sample were calculated using PAST software [40]. Differences in bacterial communities and functional group composition among the F, M and R sites were tested using OTU and functional-group abundance tables. The multivariate homogeneity of group dispersion (variance) of OTU and functional group data were first tested, using the “betadisper” function in the vegan package, on R software [41]. Both OTU and functional-group data were homogenously dispersed (*P*>0.05), allowing nonparametric multivariate analysis of variance to be performed (NPMANOVA) [42]. Subsequently, PAST software was used to test for differences in community taxonomic composition and functional group composition, among the 3 sample types, using NPMANOVA (P-value was calculated based on 999 permutations). Patterns in the data distribution (OTUs and functional groups) were identified and visualized by ordination: non-metric multidimensional scale (NMDS), based on Bray-Curtis distance (abundance data: number of sequences in each specific OTU/functional groups) and Jaccard distance (presence/absence data of OTUs/ functional groups), using PAST software. In this study, we analyzed the community composition using both relative abundance and presence/absence, as relative abundance data, received from next-generation sequencing, may not be used quantitatively [43].

#### Correlations between environmental factors and bacterial communities

Spearman rank correlation was used to detect correlations between individual soil and vegetation variables using PAST. Consequently, seven representative variables (total ground flora cover, pH (CaCl_2_), CEC, clay, sand, moisture and SOM) were selected, based on environmental characteristics and their correlation (S1 Fig). Goodness-of-fit (*R²* values) of the environmental valuables (17 soil variables and 5 plant variables) that fitted to the NMDS of bacterial community composition were calculated, using the “envfit” function in the “vegan” package of R software (P-value was tested with 999 permutations) [41]. Since many variables were correlated with the NMDS ordination (S2 Table), only representative variables were subsequently included in the NMDS plot.

## Results

### Soil physico-chemical properties and plant variables

Forest soil (F samples) was vastly superior to mine substrates (M and R) in terms of conditions conducive to bacterial proliferation. It contained significantly higher levels of SOM and clay than both the M and R samples did (*P* < 0.05), resulting in 5 times higher moisture content and much higher nutrient levels (N, P, K, Mg, Mn and B). Differences between the M and R samples were smaller. The subsoil, brought in to initiate forest restoration (R), was an improvement over the original mine-floor substrate (M). It contained considerably higher SOM, clay and moisture content (*P* < 0.05) than the base mine substrate. Sparse vegetation (grasses, herbs and shrubs) had patchily recolonized the rehabilitation plots; total cover remained well below 10%, whilst the untreated mine floor area remained completely denuded (Table 1).

### Quantity of living bacteria by culture method vs direct soil DNA extraction

Culturable bacteria were detected in all samples. The F samples yielded very high colony numbers that were too numerous to count (TNTC), however when samples were diluted, the number of bacterial colonies in this sample was 1.89x10^5^ CFU/gdw of soil (SE ±1.36 x 10^5^). Substantially lower cell numbers were detected in the M and R soils, 6.21 x 10^2^ (SE ±1.53x10^2^) and 1.11 x 10^3^ (SE ±4.33x10^2^) respectively; not significantly different (*P* > 0.05, S2 Fig).

When DNA was directly extracted from substrate samples, F soil yielded far more DNA (147.62–193.63 μg/gdw of soil) than did the M and R samples (0.53–2.21 μg/gdw of soil). When PCR inhibitor was removed from the samples (using OneStep PCR Inhibitor Removal kit from Zymo Research), no bacterial DNA (PCR products) was detected on agarose gel from the M and R samples (S3 Table). Although, we applied two other commercial soil DNA extraction kits and followed the DNA extraction protocol of Direito et al. [30] and Aoshima et al. [31], the results were consistent with the NucleoSpin® Soil DNA extraction kit: the F soil samples yielded high concentrations of DNA, whilst the M and R samples yielded low concentrations of DNA. Importantly, no PCR products were detected in M and R samples (S3 Fig and S4 Table). The inability to amplify DNA from substrate samples mandated the use of the culture-dependent method for subsequent DNA base-NGS analysis.

### Bacterial taxonomic richness and community composition

The rarefaction curves of cultured bacteria derived from each sample were gradually flat at the analyzed sequencing depth (30,000 reads per sample) reflecting that the detected OTUs were sufficiently high to represent the cultured community, captured by our method (S4 Fig).

**Fig 2:**
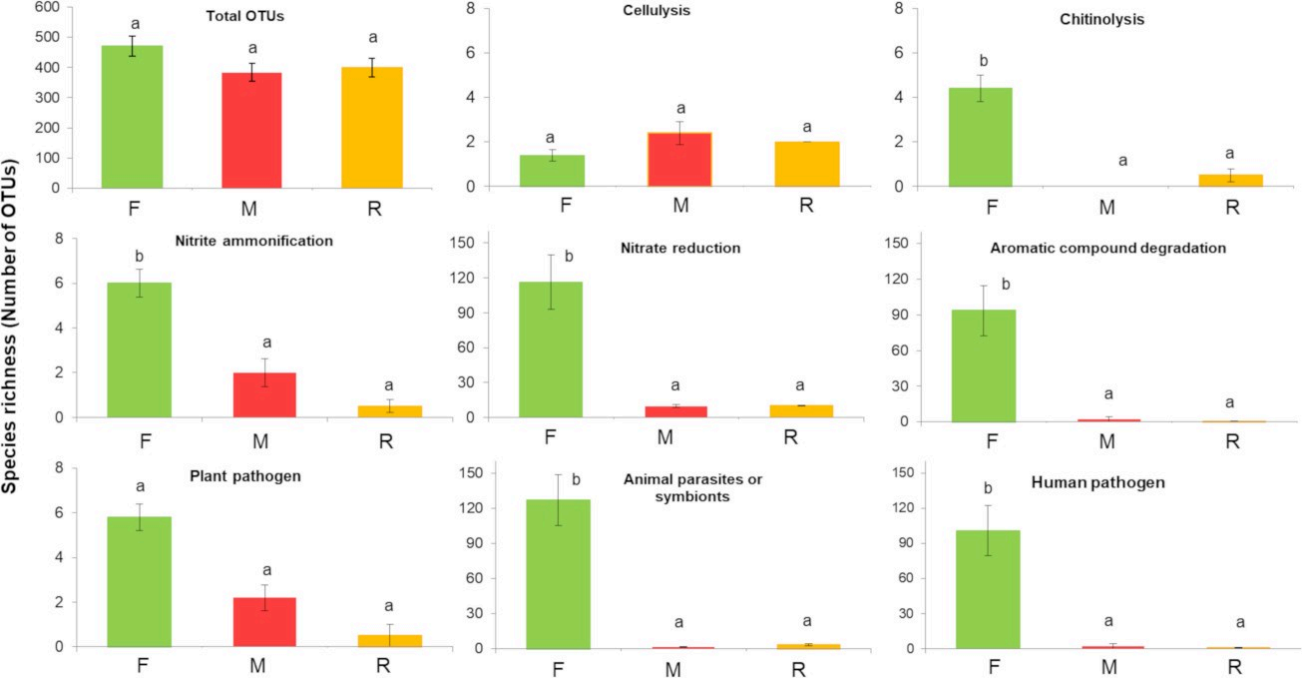
Living bacterial taxonomic richness–totals and numbers in 8 functional groups. The data derived from forest (F), Mine (M) and young mine-rehabilitation plot (R) samples. Data are numbers of OTUs (mean ±SE). Bars not sharing the same superscripts differ significantly (*P* < 0.05).

In detail, a total of 1,954 OTUs, belonging to 6 phyla, 12 classes, 20 orders, 40 families and 60 genera, were identified, across all samples and sites. Although the mine substrates supported fewer living bacteria than forest soil did; numbers of taxa, surprisingly, did not differ significantly among all 3 sample types (*P* > 0.05). The mean numbers of different OTUs, detected per sample for each site, were: F, 470 ± 33 (mean ± SE, *n* = 5), M, 384 ± 29 (*n* = 5) and R, 400 ± 30 (*n* = 4) (Fig 2). The total number of OTUs in the F, M and R samples (across all samples per site) were 1,000, 922 and 950, respectively. Similarly, 45 to 46 genera were found at each of the 3 study sites (combined across all samples).

In addition, for forest soil samples only, we compared bacterial taxonomy derived from DNA extracted directly for the soil (environmental DNA: eDNA) with that derived from cultured colonies. The method is described in detail in S1 Text. A total of 2,177 OTUs were derived from forest soil eDNA. The dominant phyla were Acidobacteria, Actinobacteria, Proteobacteria and Chloroflexi. In contrast, the cultured bacterial community in forest soil was dominated by the phyla Proteobacteria and Firmicutes. The live, culturable, bacterial community, therefore, represented a small subset of the total community. Only four out of eighteen phyla: Proteobacteria, Firmicutes, Bacteroidetes and Actinobacteria in the eDNA community could be cultured. Phyla Deferribacteres was detected only by culturing. At the genus level, 4% of bacterial communities were common to both the eDNA and culturable communities (S5 Fig).

**Fig 3:**
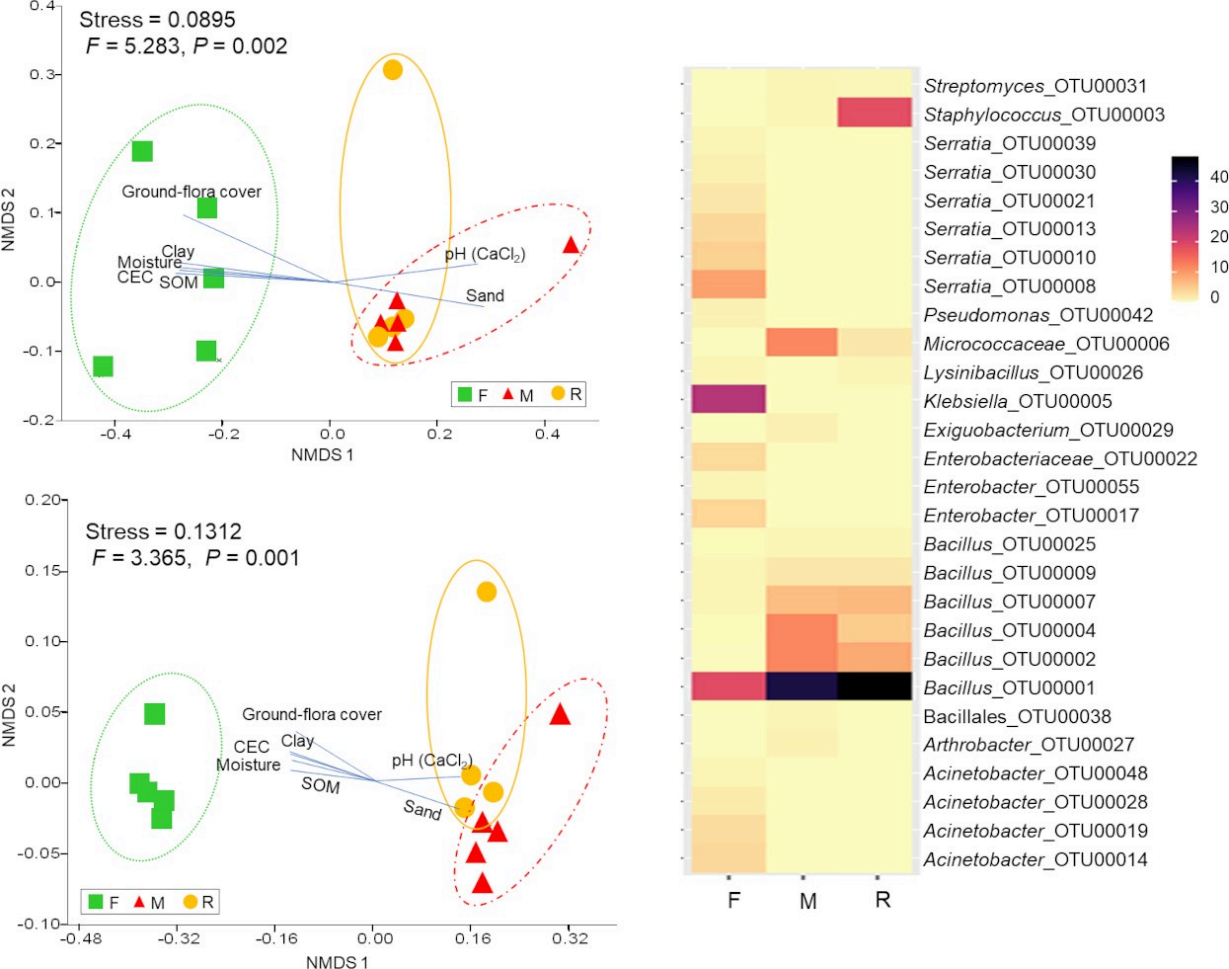
Bacterial community composition. Non-metric multidimensional scaling (NMDS) ordination of bacterial communities, under three different treatments (square: forest (F), triangle: mine (M) and circle: young mine-rehabilitation (R), based on Bray-Curtis distance (top left) and Jaccard distance (lower left). Significant representative soil physico-chemical properties and plant variables (*P* < 0.05) were plotted in the respective NMDS ordination plots. Heat-map showing the percentage of OTU relative abundance (over 0.05% of the community in each sample).

**Fig 4:**
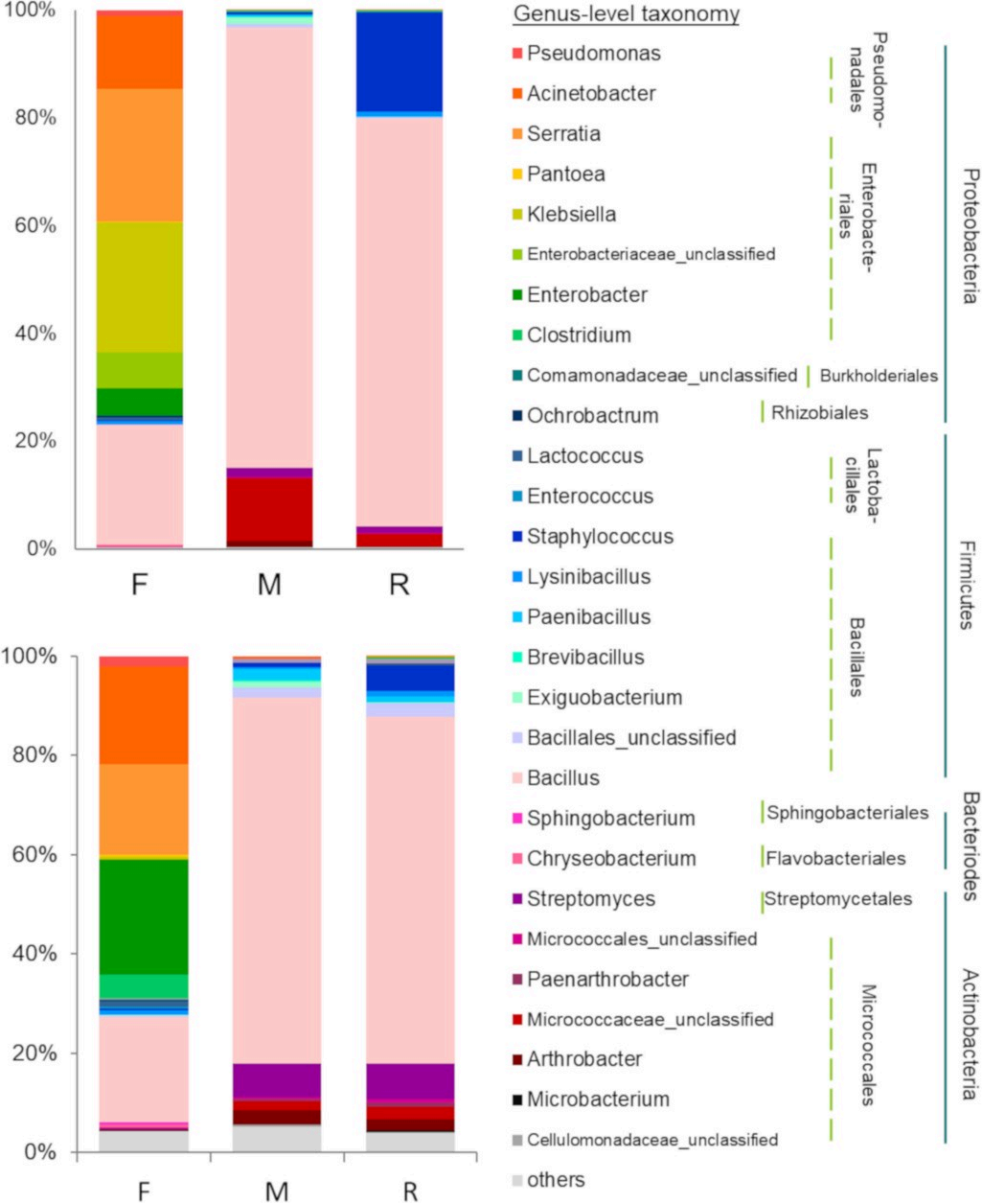
Taxonomic distribution of living bacteria. Distribution data, derived from forest (F), mine (M) and young mine-rehabilitation plot (R) samples, in terms of relative abundance (top left) and incidence (bottom left) data, at genus level (colored-boxes legend). The lines indicate the order (dotted) and phylum (solid) of bacteria detected.

The taxonomic composition of the living bacterial communities in F samples differed significantly from that in R and M samples, according to both abundance data (*F* = 5.283, *P* = 0.002) and presence/absence data (*F* = 3.365, *P* = 0.001) (Fig 3). *Klebsiella* (24.05%) was the most abundant genus in F samples, followed by *Serratia* (24.53%) and *Bacillus* (22.24%). According to abundance data, the F samples were dominated by Proteobacteria (75.69%) followed by Firmicutes (23.79%), Bacteroidetes (0.44%), Actinobacteria (0.08%) and Deferribacteres (< 0.01%). Similarly, presence/absence data revealed that more than 60% of the OTU richness belonged to Proteobacteria (Fig 4).

The taxonomic composition of the microbial communities between M and R samples did not differ significantly (Fig 3). Species of the genus *Bacillus* dominated (M = 81.73%, R = 75.79%). Firmicutes dominated the M and R samples (M = 84.95%, R = 95.79%), followed by Actinobacteria (M = 14.83%, R = 4.03%), Proteobacteria (M = 0.22%, R = 0.17%), Bacteroidetes and Deinococcus-Thermus (both about 0.02%) (Fig 4). A similar result was obtained with presence/absence data (OTU richness). More than 75% of detected OTU’s in the M and R samples were Firmicute, followed by Actinobacteria (> 15% of OTU richness, Fig 4).

### Bacterial functional groups

**Fig 5:**
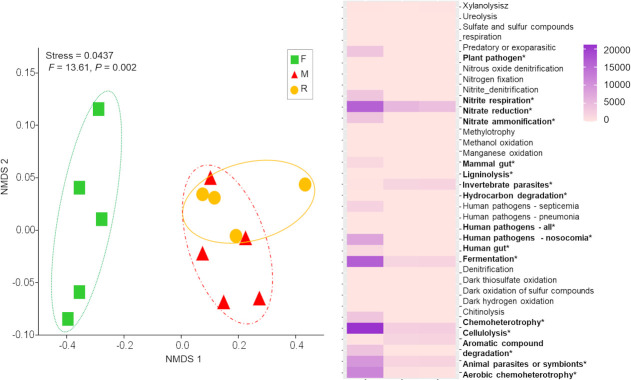
Bacterial functional groups composition. Non-metric multidimensional scaling (NMDS) ordination of bacterial functional groups under three different treatments, square: forest (F), triangle: mine (M) and circle: young mine-rehabilitation (R), based on Bray-Curtis distance. Heat-map showing the percentage of OTU abundance for individual function.

Six hundred and eleven out of 1,954 OTUs (31.3%) were assigned to at least one of the 37 functional groups detected. The functional group composition of the F bacterial communities differed significantly from that of the M and R samples (*F* = 13.61, *P* = 0.002; Fig 5). The high diversity of functional groups, found in the F samples, reflected high abundance of bacteria (high number of sequences), especially those involved in carbon and nitrogen cycling (chitinolysis, aromatic compound degradation, nitrate and nitrite respiration, ammonification and nitrate reduction) and those associated with plant-animal interactions (plant pathogens, animal pathogens and symbionts: Fig 5). Furthermore, the numbers of OTUs that performed such functions in F were significantly higher (*P* < 0.05) than those in M and R samples (Fig 2). Several of the abundant functional groups in the F samples were rare in or absent from the R and M samples (Fig 5).

### Factors corresponding with bacterial community composition and their derived functions

NMDS ordination of the bacterial community composition showed similar correlations between soil/plant variables and bacterial functional group composition. In particular, pH and sand were positively and significantly correlated with the bacterial and functional composition of both M and R samples, while community composition of F samples were positively correlated with moisture content, SOM, CEC, clay proportion, and total plant cover (Fig 3 and S2 Table).

## Discussion

The bacterial taxa, recorded in this study, may be useful for restoration purposes, since they can survive in the extreme environment of limestone mine substrates and they can be cultured easily to produce inoculae.

### Living soil bacterial analysis: Benefit and drawback

For forest soil, we demonstrated that the cultured bacteria community was different from that derived from eDNA. As expected, only a small subset of bacteria detected by eDNA were culturable, and a few taxa that were not detected by eDNA analysis did show up in cultures (see also Stefani et al. [44]). Therefore, the need to use culturable bacteria, to overcome the difficulty in extracting and amplifying DNA directly from mine substrates, was a limitation to this study. The culture media and conditions may have selectively allowed only some species to survive, whilst eliminating others. Fast-growing bacteria may have out-competed slower growing species (e.g. actinomycetes) [45]. To avoid such potential biasness, we suggest future experiments explore a greater variety of culture media, durations, and other conditions. On the other hand, the culture technique identified taxa that could be easily mass produced for soil ecosystem restoration purposes. This method revealed not only bacterial taxonomy but also ecological functionality of the detected taxa. It can be used for fast screening of living bacteria structure and their associated function in any sample type. However, notice should be taken of the limitations of next-generation sequencing: it cannot define all bacterial taxonomy to species level and some OTUs may only be identifiable to order or genus level.

### Hidden diversity of living soil bacteria in mine substrates

Our first hypothesis–that some bacteria taxa can persist in mine substrates, despite severe degradation—was validated.

Several of the living bacterial taxa that inhabited the mine substrates were the same as those found in forest soil; respectively, 23.4% and 25.9% of OTUs detected in forest samples, were found in the M and R samples. This is consistent with a previous study, which showed that soil degradation did extirpate some bacterial taxa, whilst others survived [23]. However, we also found that mining considerably reduced the living bacteria population density, compared with forest soil (based on number of bacterial colonies).

Consequently, we hypothesized differences existed in the diversity and taxonomic composition of the living bacterial communities among the soil/substrates. According to a previous study, forest soil was expected to support greater taxonomic richness than the two mine substrates [24]. Differences in community taxonomic composition between forest soil and both mine substrates, were indeed significant, although differences in taxonomic richness were not. The taxonomic richness of living bacterial OTUs was similar at all 3 sampling sites. We postulate that the species richness of forest soils and mine substrates was similar because culturable bacterial species survived the harsh conditions of mining by reducing their numbers (abundance) while maintaining the bacterial diversity. This contradicts other studies, based on direct analysis of soil DNA (i.e. no preculturing), which reported low bacterial diversity in mine substrates and early rehabilitation soils, compare to undisturbed sites [15, 46]. It should be noted that use of eDNA to determine taxonomic richness includes both living and dead organisms [47, 48], which results in more species being detected. Furthermore, the selectivity of the culturing methods, used in our study, most probably resulted in lower taxonomic richness being recorded in forest soil, thus resulting in similar taxonomic richness of living bacteria between forest soil and mine substrates. This assumption could be tested by increasing the incubation time and varying the culture media composition. However, our study confirmed that many living bacteria groups are capable of surviving under the extreme conditions found in mine substrates and thus provides important information for the development of effective mine rehabilitation practices.

Although species richness of living bacteria in mine substrates was high, their population density was low. The close proximity of the mine to surrounding intact forest, might explain the arrival of large numbers of bacteria species on the mine (via spore dispersal), whilst the poor substrate conditions might explain why they subsequently failed to proliferate as much as forest soil bacteria did.

The similarity in taxonomic composition of the living bacterial communities of the M and R samples implied that nine months was not enough time for the planted tree seedlings and sparse ground flora in the R site to ameliorate the micro-habitat conditions enough to differ the bacterial species present from M site. Since R substrate was an overburden left after mining operation, most physico-chemical properties including pH, micronutrients and macronutrients, were not different from M substrate. Although organic matter and moisture content of the R samples were significantly higher than those of the M samples, the difference was small and may not have been attributable to the rehabilitation operations, since the original condition of the material, brought in before tree planting, was unknown. However, this study showed that slight differences in organic matter and moisture content between M and R samples were not impacts living bacterial community composition. Previous studies have demonstrated that long-term revegetation of mines improves soil fertility, which facilitates bacterial growth, taxonomic diversity and changes community composition [15, 49].

Aislabie and Deslippe [11] reported that the most abundant bacteria in soil samples are Proteobacteria. Our results from the F samples support this view, whereas those from the M and R samples did not. Firmicutes, especially *Bacillus*, dominated the M and R samples, followed by Actinobacteria (*Streptomyces* and *Arthrobacter*). This may have been due to the harsh conditions in the mine area, including dryness and elevated exposure to heat and UV irradiation. Firmicutes (*Bacillus*) and Actinobacteria are known to thrive in such extreme environments, by producing spores that render them resistant to environmental stress [50, 51].

### Lack of functional redundancy in mine substrate and young mine-rehabilitation soil

Our work agrees with that of Atlas et al. [52], who reported that high microbial diversity does not always result in high functional redundancy (i.e. many bacterial taxa performing the same ecological functions). Although, the taxonomic richness of the R and M samples was as high as that of the F samples, functional redundancy of the R and M samples was significantly lower–a condition regarded as ecologically less stable [53]. To the best of our knowledge, few studies of functional redundancy of microbiota along a chronosequence of mine reclamation have been published. Our work showed that functional redundancy, related to nitrogen and carbon cycles in mine substrates, is considerably lower than that of forest soil. Furthermore, we showed that functional redundancy remained as low as that of mine-floor substrates, over at least the first 9 months of rehabilitation operations. This indicates that restoration of microbial functional in mine substrates takes several years. Yin et al. [24] also reported low functional redundancy within bacterial communities of mine substrates, but that it increased when plant communities were re-established. This can be explained by inputs of organic matter into the substrate via litter fall and plant death that widen niches, consequently allowing populations of various functionally redundant taxa to recover. However, since the literature on this topic is so sparse, further studies of the recovery of functional redundancy of microbial communities as mine rehabilitation proceeds are needed.

### Correlation of soil and plant variables with community taxonomic and functional composition

Our work confirms that mine substrates have extremely poor physico-chemical properties (low organic matter, nutrients and poor moisture retention), compared with forest soil [54]. Although other studies used a top-soil stockpile to recondition the mine floor before rehabilitation, in our study site, loosened sub-soil was used (R). After 9 months of rehabilitation, this substrate supported better plant re-establishment, compared with the original mine-floor substrate (M) (Fig 1). Per cent clay, organic matter and soil moisture in the R samples were significantly higher than in the M samples. This might be explained by lack of disturbance of the substrate for 9 months and proximity to nearby seed sources, resulting in re-establishment of around 8–10% vegetation cover. However, improvements in conditions of the R samples were too small to have had a substantial impact on the soil bacteria. Li et al. [15] demonstrated that bacterial community composition altered along a chronosequence of mine rehabilitation, and that it took about 15–20 years for the bacterial community structure of mine rehabilitation plots to match that of undisturbed areas. We showed that the taxonomic composition of the living bacterial community hardly changed after 9 months of rehabilitation. Correlation between soil properties was tested by Spearman rank correlation. We demonstrated soil water-holding capacity, organic matter, CEC and soil nutrients decreased with increasing sand proportion, which was consistent with the findings of Walpola and Arunakumara [55]. Moreover, we showed that those physico-chemical variables correlated with both the taxonomic and functional composition of the bacterial communities, and they may have been the cause of the differences in the latter between forest soil and mine. In particular, soil moisture, organic matter and soil nutrients significantly affect microbial activities [56]. Low levels of these properties limit the growth of some bacteria. In this study, Firmicutes were abundant in the M and R samples, which had low levels of organic matter and water content in agreement with the results of Van Horn et al. [56] and Van Horn et al. [57]. Conversely, the abundance of Proteobacteria in the F sample could be explained by high organic content and nutrient availability, also in agreement with previous studies [58, 59].

### Applications for mine rehabilitation

The material brought on to the mine, to support tree planting was nutrient-poor with low levels of organic matter and moisture retention. Consequently, the physico-chemical properties of this material did not support microbial proliferation, as it contained considerably lower amounts of living bacteria relative to forest soil. We suggest that the addition of organic matter, to increase moisture retention, would most likely ameliorate the condition of the substrate and, if the organic matter were to be derived from forest soil or leaf litter, it would also act as an inoculum of all the bacteria taxa needed to kick-start restoration of soil ecosystem functioning [60–62]. Moreover, addition of other carbon sources, such as agricultural waste, could also encourage rapid bacterial growth [62, 63]. However, caution should be exercised, to ensure that the organic materials used do not include or promote the production of invasive microorganisms and do not contains seeds of invasive exotic plant species. For example Castillejo and Castelló [64] reported that organic amendment can encourage the development of an introduced plant species and suppressed that of native species. If the objective is restoration of indigenous forest ecosystems, then obviously, it makes sense that any such added organic matter or soil should come from intact old-growth forest of the target forest ecosystem type. Wubs et al. [65] showed that inoculation of topsoil from late-successional ecosystems can both improve soil properties and direct ecological succession towards the condition of the donor site. Forest soil not only has relatively high organic matter content, which ensures high moisture-holding capacity and nutrient availability, but it also contains microbial inoculum and a plant seed bank which facilitate natural ecological succession [66]. Therefore, use of forest soil, rather than agricultural waste, is recommended.

If mine rehabilitation procedures include microbial inoculation, then taxa that can survive in the harsh conditions of mine substrates should be used [18]. We demonstrated that the genera *Bacillus*, *Streptomyces* and *Arthrobacter* thrive in mine substrates and they can be cultivated easily. Thus, inoculation of these taxa to support rehabilitation is of particular interest. Further studies to test the drought tolerance of these species and their plant growth-promoting ability are recommended. Bacterial community composition should be monitored over several years post-rehabilitation.

## Supporting information

S1 Fig Spearman’s rank correlations among individual soil and plant parameters. Bold numbers indicate significant correlations (*P* < 0.05). Highly correlated factors (*R* > 0.70 or *R* < -0.70, *P* < 0.01) were highlighted with pink color.
(TIF)

S2 Fig Bacterial colonies presented on three different media. Average CFU’s (mean ± SE) found in forest (F), mine (M) and young mine-rehabilitation plots (R) samples. Negative controls are also shown in the bottom line. Values not sharing the same superscript are significantly different (*P* < 0.05).
(TIF)

S3 Fig Agarose gel of PCR product show availability of soil bacteria. The results from direct DNA extraction of Forest soil (F), mine substrate (M), and young mine-rehabilitation substrate (R).
(TIF)

S4 Fig Sample-based rarefaction curve of observed bacterial OTU for soil/substrate samples. A) Forest soil, B) Mine substrate and C) Rehabilitation substrate.
(TIF)

S5 Fig Krona charts showing the taxonomic identification and occurrence of bacteria derived from soil direct DNA extraction and cultured media. The proportion of bacterial taxa derived from A) culture media and B) directed DNA extraction (eDNA). Yellow star indicted shared taxa between culture media and directed DNA extraction. C-F) Dominant phyla detected by directed DNA extraction.
(TIF)

S1 Table Protocol for soil physicochemical analysis. (PDF)

S2 Table Goodness-of-fit statistics (*R*^*2*^) and P-value of environmental variables. Fitted to the non-metric multidimensional scaling (NMDS) ordination of bacterial community composition and functional groups.
(PDF)

S3 Table Quality and quantity of DNA which directly extracted from soil/substrate. DNA Concentration, purity and the availability of bacterial DNA detected on agarose gel.
(PDF)

S4 Table Summary of PCR results derived from 9 different protocols. (PDF)

S1 Text Method for the comparison of bacterial taxonomy derived from direct soil DNA extraction and culture media. (PDF)
