# Supplementary figures and images for: Soil bacterial communities and their associated functions for forest restoration on a limestone mine in northern Thailand
Source: PLoS One. 2021 Apr 8;16(4):e0248806. doi: 10.1371/journal.pone.0248806 (PMC8031335; doi:10.1371/journal.pone.0248806)

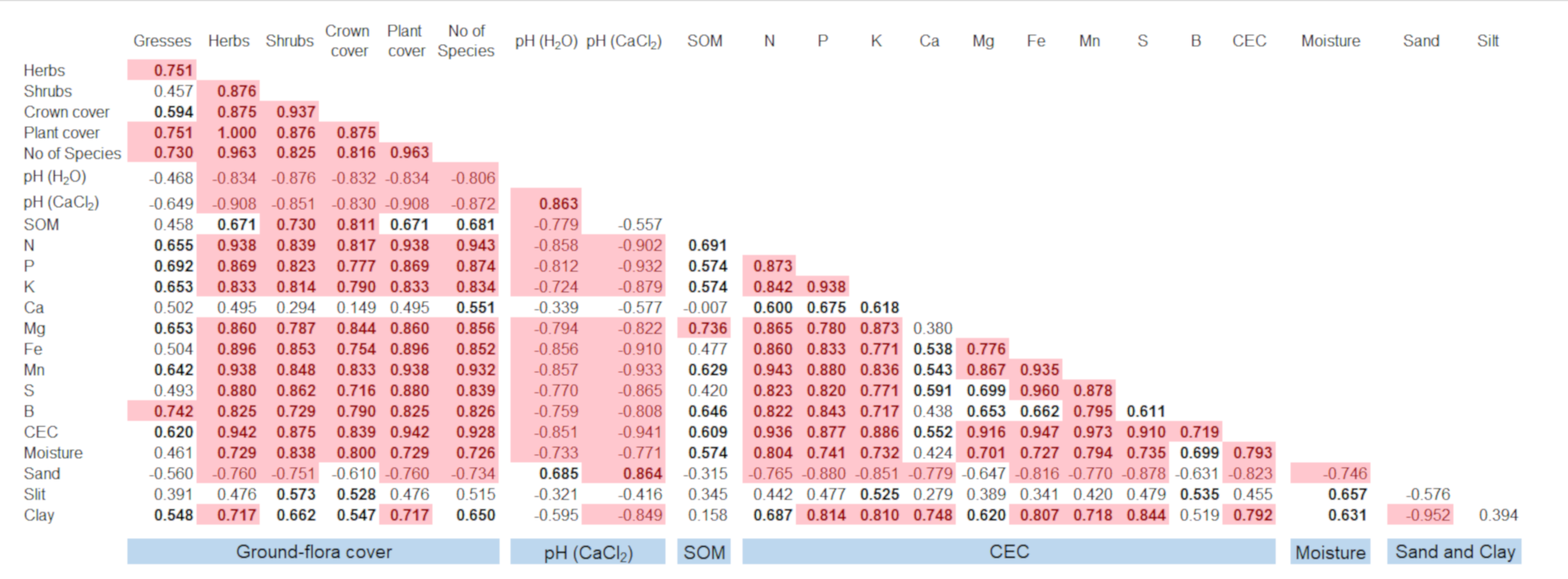

Supplement: S1 Fig — Bold numbers indicate significant correlations (P < 0.05). Highly correlated factors (R > 0.70 or R < -0.70, P < 0.01) were highlighted with pink color. (TIF) [file pone.0248806.s001.tif]

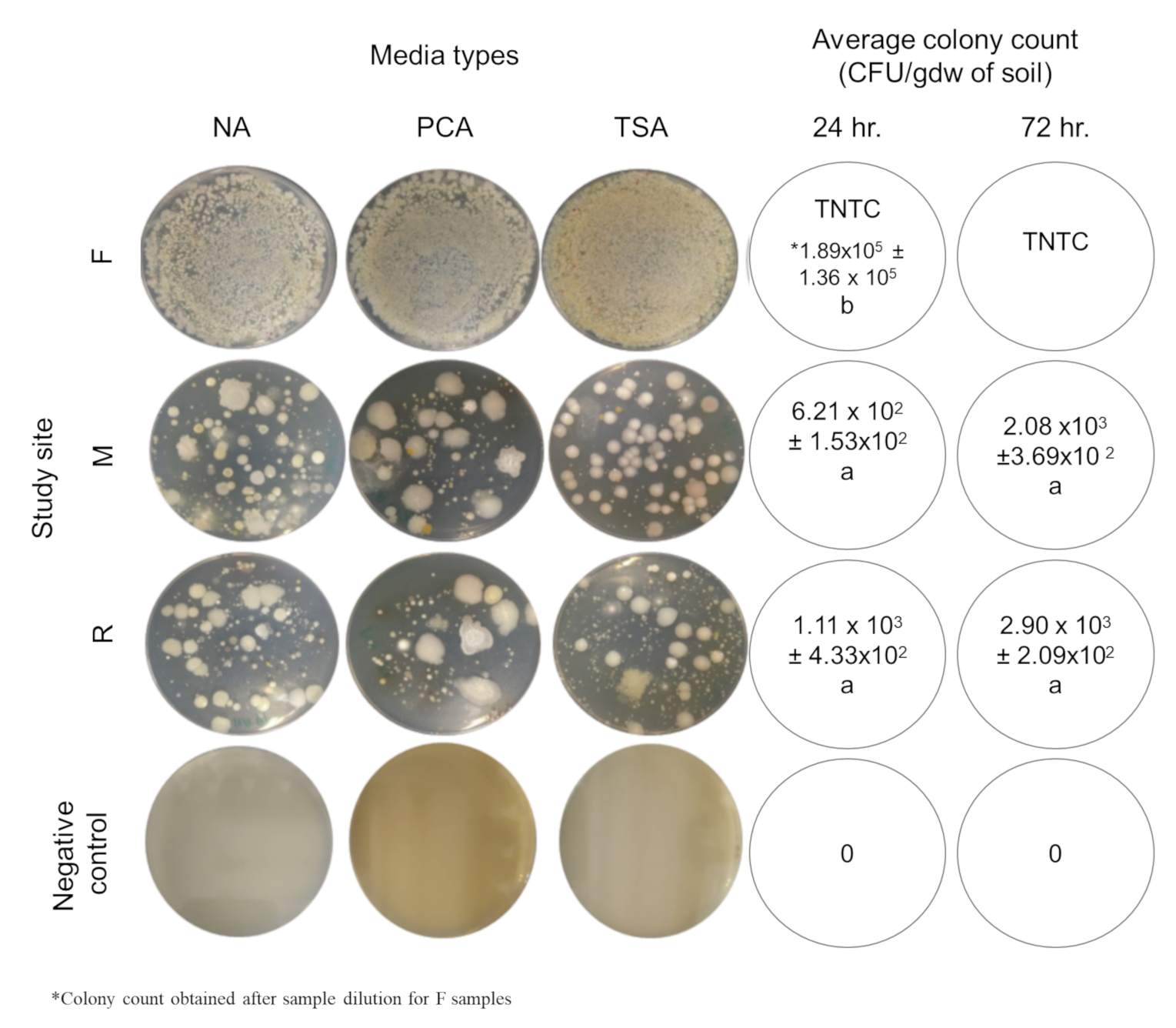

Supplement: S2 Fig — Average CFU’s (mean ± SE) found in forest (F), mine (M) and young mine-rehabilitation plots (R) samples. Negative controls are also shown in the bottom line. Values not sharing the same superscript are significantly different (P < 0.05). (TIF) [file pone.0248806.s002.tif]

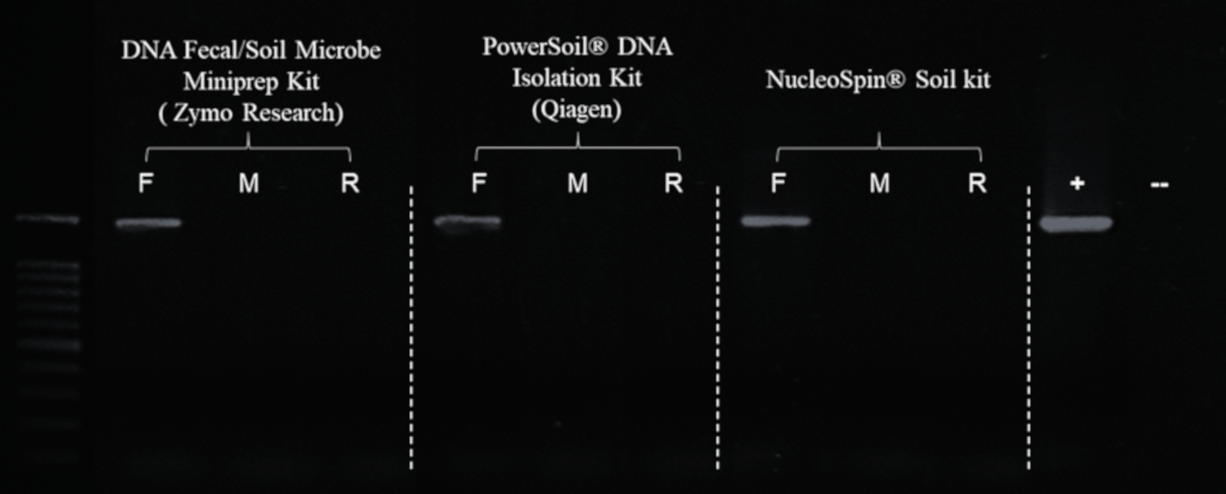

Supplement: S3 Fig — The results from direct DNA extraction of Forest soil (F), mine substrate (M), and young mine-rehabilitation substrate (R). (TIF) [file pone.0248806.s003.tif]

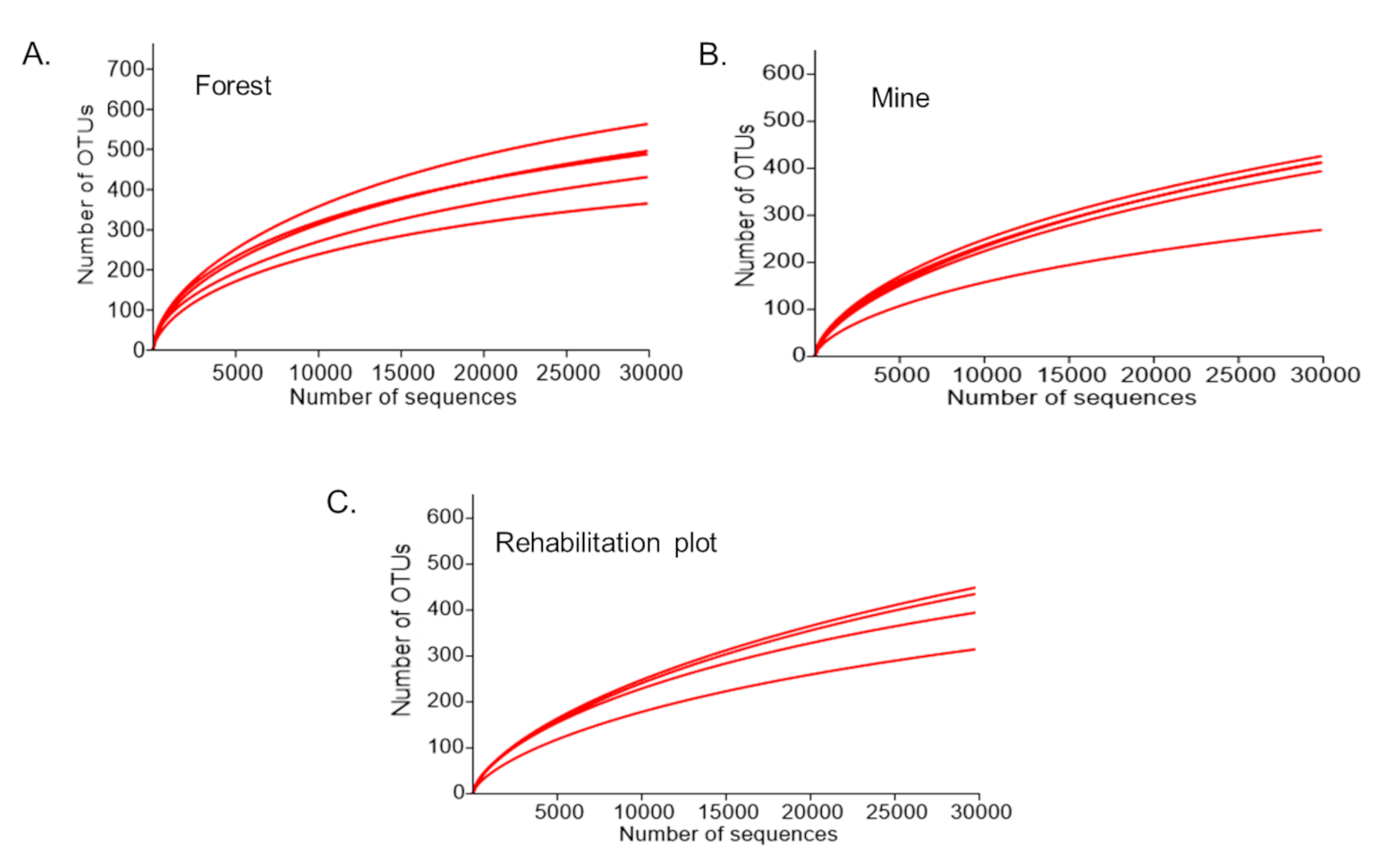

Supplement: S4 Fig — A) Forest soil, B) Mine substrate and C) Rehabilitation substrate. (TIF) [file pone.0248806.s004.tif]

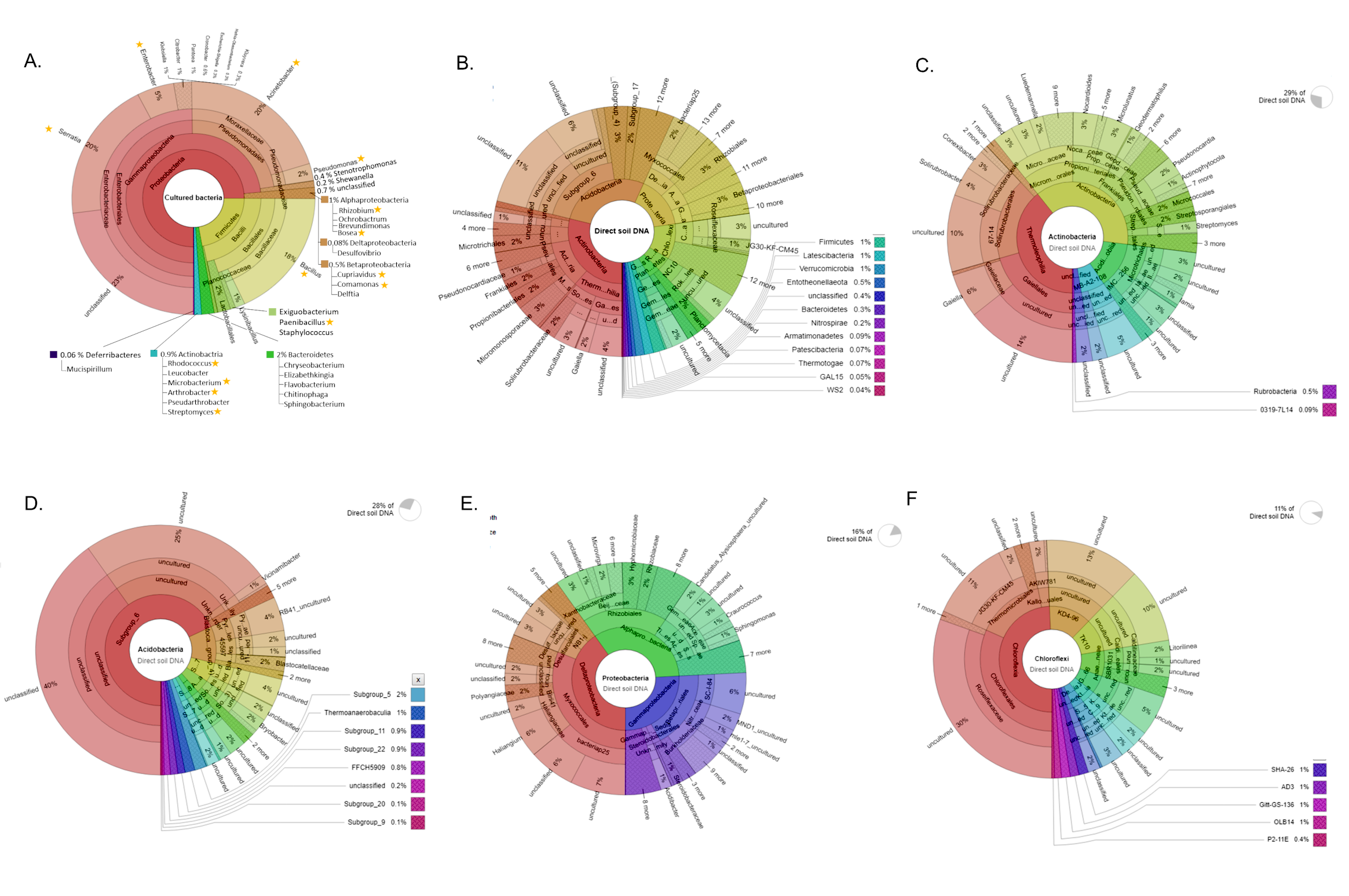

Supplement: S5 Fig — The proportion of bacterial taxa derived from A) culture media and B) directed DNA extraction (eDNA). Yellow star indicted shared taxa between culture media and directed DNA extraction. C-F) Dominant phyla detected by directed DNA extraction. (TIF) [file pone.0248806.s005.tif]
