# Supplementary material for: Soil bacterial communities and their associated functions for forest restoration on a limestone mine in northern Thailand
Source: PLoS One. 2021 Apr 8;16(4):e0248806. doi: 10.1371/journal.pone.0248806 (PMC8031335; doi:10.1371/journal.pone.0248806)
Supplement: S1 Table — (PDF) [file pone.0248806.s006.pdf]

S1 Table. Protocol for soil physicochemical analysis

Soil parameters	Protocols
pH(H2O)	Rayment & Higginson (1992)
pH(CaCl2)	Rayment & Higginson (1992)
Soil Organic Matter (SOM)	Walkley-Black method
Total nitrogen (N)	Kjeldahl method
Available Phosphorous (P)	Bray II extraction
Exchangeable Potassium (K)	NH4OAC pH7 method/ Atomic Emission Spectroscopy (AES)
Exchangeable Calcium (Ca)	NH4OAC pH7 method/ Atomic Absorption Spectroscopy (AAS)
Exchangeable Magnesium (Mg)	NH4OAC pH7 method/ Atomic Absorption Spectroscopy (AAS)
Extractable Sulfur (S)	$BaCl_2$ method
Available Iron (Fe)	DNTP/ AAS
Available Manganese (Mn)	DNTP/ AAS
Exchangeable Boron (B)	$CaCl_2$ mannitol extraction
Cation Exchange Capacity (CEC)	NH4OAC pH7/ Distillation
moisture	Oven-drying method
sand	Sieve/Hydrometer
slit	Sieve/Hydrometer
clay	Sieve/Hydrometer
