# Supplementary material for: Soil bacterial communities and their associated functions for forest restoration on a limestone mine in northern Thailand
Source: PLoS One. 2021 Apr 8;16(4):e0248806. doi: 10.1371/journal.pone.0248806 (PMC8031335; doi:10.1371/journal.pone.0248806)
Supplement: S2 Table — Fitted to the non-metric multidimensional scaling (NMDS) ordination of bacterial community composition and functional groups. (PDF) [file pone.0248806.s007.pdf]

S2 Table. Goodness-of-fit statistics (R^2) and *P*-value of environmental variables. Fitted to the non-metric multidimensional scaling (NMDS) ordination of bacterial community composition and functional groups

Environmental parameters	Community composition				Functional groups with Bray-Curtis distance	
	Bray-Curtis		Jaccard			
	R^2	*P*	R^2	*P*	R^2	*P*
**Soil parameters**						
**pH(H_2O)**	0.93	**0.001***	0.93	**0.001***	0.92	**0.001***
**pH($CaCl_2$)**	0.92	**0.001***	0.92	**0.001***	0.92	**0.001***
**Moisture (%)**	0.98	**0.002***	0.98	**0.001***	0.98	**0.001***
**SOM (%)**	0.99	**0.002***	0.99	**0.001***	0.99	**0.001***
**Total N (%)**	0.99	**0.002***	0.99	**0.001***	0.99	**0.001***
**P (mg/kg)**	0.50	**0.032***	0.50	**0.035***	0.50	**0.022***
**K (mg/kg)**	0.78	**0.003***	0.78	**0.002***	0.78	**0.001***
**Ca (mg/kg)**	0.02	0.858	0.02	0.864	0.06	0.692
**Mg (mg/kg)**	0.76	**0.002***	0.76	**0.002***	0.75	**0.001***
**Fe (mg/kg)**	0.68	**0.007***	0.68	**0.004***	0.68	**0.005***
**Mn (mg/kg)**	0.71	**0.002***	0.71	**0.001***	0.71	**0.001***
**S (mg/kg)**	0.20	**0.287***	0.19	**0.285***	0.18	0.302
**B (mg/kg)**	0.98	**0.001***	0.98	**0.001***	0.98	**0.001***
**CEC (cmol/kg)**	0.92	**0.001***	0.92	**0.001***	0.91	**0.001***
**Sand (%)**	0.90	**0.003***	0.90	**0.001***	0.89	**0.001***
**Slit (%)**	0.22	0.228	0.22	0.220	0.22	0.223
**Clay (%)**	0.93	**0.002***	0.93	**0.001***	0.93	**0.001***
**Plant parameters**						
**Grasses (%)**	0.56	**0.006***	0.56	**0.010***	0.55	**0.007***
**Herbs (%)**	0.84	**0.003***	0.84	**0.001***	0.84	**0.003***
**Shrubs (%)**	0.87	**0.003***	0.87	**0.001***	0.87	**0.001***
**Total ground cover (%)**	0.82	**0.002***	0.82	**0.002***	0.85	**0.001***
**Tree crown cover (%)**	0.82	**0.003***	0.82	**0.001***	0.82	**0.001***
**No. of plant species**	0.80	**0.001***	0.80	**0.001***	0.77	**0.003***
