# Supplementary material for: Soil bacterial communities and their associated functions for forest restoration on a limestone mine in northern Thailand
Source: PLoS One. 2021 Apr 8;16(4):e0248806. doi: 10.1371/journal.pone.0248806 (PMC8031335; doi:10.1371/journal.pone.0248806)
Supplement: S3 Table — DNA Concentration, purity and the availability of bacterial DNA detected on agarose gel. (PDF) [file pone.0248806.s008.pdf]

S3 Table. Quality and quantity of DNA which directly extracted from soil/substrate. DNA Concentration, purity and the availability of bacterial DNA detected on agarose gel.

Study sites	Samples	Concentration (µg/gdw of soil)	Purity (A260/A280)	Available of Bacterial DNA/amplicon results on agarose gel*
Forest	F1	147.62	1.91	clear band
	F2	186.48	1.64	clear band
	F3	138.94	1.8	clear band
	F4	161.47	1.9	clear band
	F5	193.63	1.93	clear band
Mine	M1	1.17	1.1	no band
	M2	2.12	1.67	no band
	M3	1.58	1.26	no band
	M5	2.07	1.38	no band
	M6	1.08	1.29	no band
Rehabilitation	P1	2.21	1.42	no band
	P3	0.53	0.84	no band
	P4	2.14	1.67	no band
	P6	0.71	1.43	no band

*Note: Clear band: presence of bacterial DNA in a sample, No band: absence of bacterial DNA in a sample
