# Supplementary material for: Soil bacterial communities and their associated functions for forest restoration on a limestone mine in northern Thailand
Source: PLoS One. 2021 Apr 8;16(4):e0248806. doi: 10.1371/journal.pone.0248806 (PMC8031335; doi:10.1371/journal.pone.0248806)
Supplement: S4 Table — (PDF) [file pone.0248806.s009.pdf]

S4 Table. Summary of PCR results derived from 9 different protocols.

DNA Extraction protocol	PCR result		
	Forest	Mine	Rehabilitation
NucloSpin® Soil kit + zymo PCR removal kit	Clear band	No band	No band
DNA Fecal/Soil Microbe Miniprep kit	Clear band	No band	No band
PowerSoil ®DNA Isolation kit	Clear band	No band	No band
DNA extraction protocol by Aoshima et al. (2005)	Clear band	No band	No band
NucloSpin® Soil kit + zymo PCR removal kit + OneStep PCR Inhibitor Removal kit	Clear band	No band	No band
DNA Fecal/Soil Microbe Miniprep kit + OneStep PCR Inhibitor Removal kit	Clear band	No band	No band
PowerSoil ®DNA Isolation kit + OneStep PCR Inhibitor Removal kit	Clear band	No band	No band
DNA extraction protocol by Aoshima et al. (2006) [1]*+ OneStep PCR Inhibitor Removal kit	Clear band	No band	No band
DNA extraction protocol by Direito et al (2012) [2]*	Clear band	No band	No band

*Note: References for DNA extraction protocol

1. Aoshima H, Kimura A, Shibutani A, Okada C, Matsumiya Y, Kubo M. Evaluation of soil bacterial biomass using environmental DNA extracted by slow-stirring method. Appl Microbiol Biotechnol. 2006;71: 875–880. doi:10.1007/s00253-005-0245-x

2. Direito SOL, Marees A, Röling WFM. Sensitive life detection strategies for low-biomass environments: optimizing extraction of nucleic acids adsorbing to terrestrial and Mars analogue minerals. FEMS Microbiology Ecology. 2012;81: 111–123. doi:10.1111/j.1574-6941.2012.01325.x
