# Supplementary material for: Soil bacterial communities and their associated functions for forest restoration on a limestone mine in northern Thailand
Source: PLoS One. 2021 Apr 8;16(4):e0248806. doi: 10.1371/journal.pone.0248806 (PMC8031335; doi:10.1371/journal.pone.0248806)
Supplement: S1 Text — (PDF) [file pone.0248806.s010.pdf]

S1 Text. method for the comparison of bacterial taxonomy derived from direct soil DNA extraction (eDNA) and cultured media.

DNA was extracted from 0.25 g of forest soil using a NucleoSpin® Soil DNA extraction kit following the manufacturer's instructions. Then, PCR amplification of the bacterial V3-V4 region was conducted using the forward primer: Bact341F (5'-CCTACGGGNGGCWGCAG-3') and the reverse primer: Bact785R (5'-GACTACHVGGGTATCTAATCC-3') [1]. Amplification was performed in the following PCR mixture (25 μL): 22 μL of Qiagen HotStar Taq master mix (Qiagen Inc, Valencia, California), 1 μL of each 5 μM primer, and 1 μL of template. The PCR reaction was performed as follows: 95°C for 15 min, then 35 cycles of 94°C for 30 sec, 55°C for 30 sec, 72°C for 1 min, followed by one cycle of 72°C for 10 min. The PCR amplicons were then cleaned up and prepared for sequencing. Amplicons were sequenced using an Illumina MiSeq platform. All amplification and sequencing steps were performed at RTL Genomics (Lubbock, TX, USA).

Bioinformatics analysis of samples from direct soil DNA extraction was analysed on MOTHUR 1.33.3 [2] using similar parameters as cultured bacteria presented in method section of main manuscript. Briefly, raw reads were first assembled to generate paired-end reads. Pair-ending read was filtered to get high quality read using parameters as follow: A minimum 200 base pair and a minimum average quality of 30 Phred score. Chimeric sequences were detected using the UCHIME algorithm [3], as implemented in MOTHUR, and removed them from the datasets. The cleaned sequences were clustered at 97% sequence identity, then assigned taxonomy using the SILVA 16S rRNA sequence database version 128 [4]. Rare OTUs, including singletons, doubletons, and tripletons, were removed to eliminate potential sequencing errors. This dataset was rarefied to 12269 reads per sample (smallest read per sample derived from directed soil DNA

extraction). To compare the community forest soil derived from direct soil DNA extraction (eDNA) and cultured media. The dataset of cultured bacteria (result received after rare OTUs removal) was also rarefied to 12269 reads per sample.

References

1. Klindworth A, Pruesse E, Schweer T, Peplies J, Quast C, Horn M, et al. Evaluation of general 16S ribosomal RNA gene PCR primers for classical and next-generation sequencing-based diversity studies. Nucleic Acids Res. 2013;41: e1. doi:10.1093/nar/gks808.

2. Schloss PD, Westcott SL, Ryabin T, Hall JR, Hartmann M, Hollister EB, et al. Introducing mothur: open-source, platform-independent, community-supported software for describing and comparing microbial communities. Appl Environ Microbiol. 2009;75: 7537–7541. doi:10.1128/AEM.01541-09

3. Edgar RC, Haas BJ, Clemente JC, Quince C, Knight R. UCHIME improves sensitivity and speed of chimera detection. Bioinformatics. 2011;27: 2194–2200. doi:10.1093/bioinformatics/btr381.

4. Pruesse E, Quast C, Knittel K, Fuchs BM, Ludwig W, Peplies J, et al. SILVA: a comprehensive online resource for quality checked and aligned ribosomal RNA sequence data compatible with ARB. Nucleic Acids Res. 2007;35: 7188–7196. doi:10.1093/nar/gkm864.
